# A three-gene signature for prognosis in patients with MGMT promoter-methylated glioblastoma

**DOI:** 10.18632/oncotarget.11726

**Published:** 2016-08-31

**Authors:** Wen Wang, Lu Zhang, Zheng Wang, Fan Yang, Haoyuan Wang, Tingyu Liang, Fan Wu, Qing Lan, Jiangfei Wang, Jizong Zhao

**Affiliations:** ^1^ Department of Neurosurgery, The Second Affiliated Hospital of Soochow University, Suzhou, China; ^2^ Department of Neurosurgery, Beijing Tiantan Hospital, Capital Medical University, Beijing, China; ^3^ Department of Molecular Neuropathology, Beijing Neurosurgical Institute, Capital Medical University, Beijing, China; ^4^ Brain Tumor Center, Beijing Institute for Brain Disorders, Beijing, China; ^5^ Department of Ophthalmology, School of Medicine, Shandong University, Jinan, China; ^6^ Department of Neurosurgery, Zhujiang Hospital, Southern Medical University, Guangzhou, China; ^7^ Chinese Glioma Cooperative Group (CGCG), Beijing, China; ^8^ China National Clinical Research Center for Neurological Diseases, Beijing, China

**Keywords:** signature, MGMT, prognosis, glioblastoma, RNA-Seq

## Abstract

Glioblastoma is the most malignant tumor and has high mortality rate. The methylated prompter of MGMT results in chemotherapy sensitivity for these patients. However, there are still other factors that affected the prognosis for the glioblastoma patients with similar MGMT methylation status. We developed a signature with three genes screened from the whole genome mRNA expression profile from Chinese Glioma Genome Atlas (CGGA) and RNAseq data from The Cancer Genome Atlas (TCGA). Patients with MGMT methylation in low risk group had longer survival than those in high risk group (median overall survival 1074 vs. 372 days; P = 0.0033). Moreover, the prognostic value of the signature was significant difference in cohorts stratified by MGMT methylation and chemotherapy (P=0.0473), while there is no significant difference between low and high risk group or unmethylated MGMT patients without chemotherapy. Multivariate analysis indicated that the risk score was an independent prognosis factor (P = 0.004). In conclusion, our results showed that the signature has prognostic value for patients with MGMT promoter-methylated glioblastomas based on bioinformatics analysis.

## INTRODUCTION

Gliomas are the most common and lethal primary tumors of central nervous system [1]. Glioblastoma (GBM) is the most malignant tumor and has high mortality rate despite maximal tumor resection with concomitant chemoradiotherapy. It is reported that the median survival for GBM patients remained about 14 months [2].

Many biomarkers, such as mutations of PTEN, IDH1, TP53 and methylation of O(6)-methylguanine DNA methyltransferase (MGMT) promoter have been identified for GBM. PTEN is a tumor suppressor and mutation of PTEN could up-regulated the AKT pathway which play a critical role in cell cycle regulation, apoptosis, and cell migration in GBM [3]. The mutation of IDH1, which may alter DNA methylation patterns in GBM [4], may serve as an early driving mutation of GBM [5]. The methylated prompter of MGMT causes an impaired ability for cells to recover from damage induced by chemotherapeutic agents and results in chemotherapy sensitivity for GBM patients [3, 6, 7]. However, due to variable overall survival of similar MGMT methylation status, there are still other factors that affect the prognosis for GBM patients with MGMT promoter-methylated.

In our study, we obtained whole genome mRNA expression profiling microarray data from Chinese Glioma Genome Atlas (CGGA) as training set and two validation datasets, CGGA mRNA-sequencing data and The Cancer Genome Atlas (TCGA) mRNA sequencing data. By applying significance analysis of microarray (SAM) and Cox regression analysis, we screened three target genes and thus generated a signature based on these genes. Patients with MGMT promoter-methylated were divided into low risk and high risk group based on the cutoff (median risk score) and the clinical outcomes and molecular features were quite different between two groups.

## RESULTS

### A three-gene prognostic signature identified and validated in three datasets

**Figure 1:**
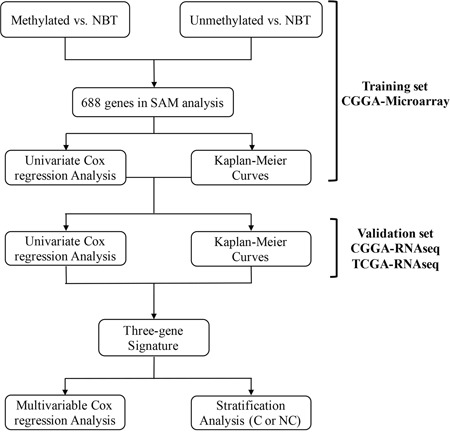
Flow chart indicating the process used to select target genes included in the analysis

A total of 41 GBM with MGMT promoter methylation, 60 GBM with MGMT promoter unmethylation and 5 non-cancerous brain tissue (NBT) samples were included in the comparison, respectively. Moreover, we compared NBT and methylated or unmethylated patients using SAM analysis and 688 mRNAs (760 probes) were differentially expressed in these samples (false discovery rate, FDR < 0.05) (Supplementary Table S1). We evaluated the prognostic value of the 688 mRNAs in training set with a univariate Cox regression analysis. Finally, three genes (FPR3, IKBIP and S100A9) were significantly associated with overall survival (OS) (Figure 1).

**Figure 2:**
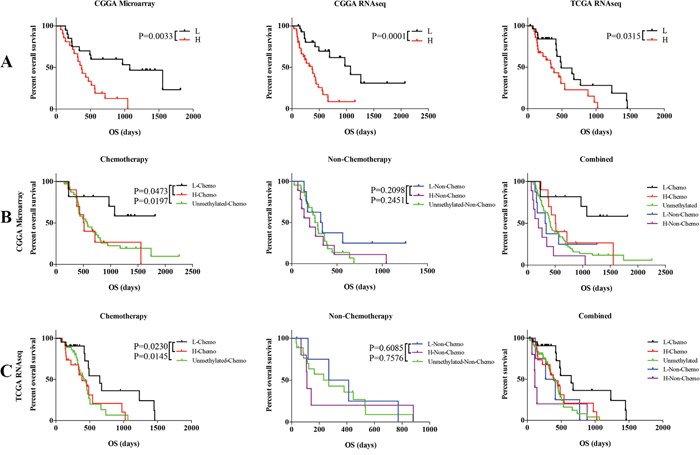
Comparison of prognosis between low and high risk group with MGMT promoter methylation GBM patients and unmethylated GBM patients **A.** Survival among GBM patients in different groups stratified by low and high risk group in three datasets. **B.** Survival among GBM patients in different groups stratified by the signature and chemotherapy in CGGA microarray dataset. **C.** Survival among GBM patients in different groups stratified by the signature and chemotherapy in TCGA RNA sequencing dataset. *P < 0.05, **P < 0.01, ***P < 0.001.

With the three genes, we developed a risk score for each patient which was calculated based on a linear combination of the mRNA expression level weighted by the regression coefficient (β) derived from the univariate Cox regression analysis. Patients with MGMT promoter methylated (41 patients) in the training set were divided into low risk and high risk group based on the cutoff (median risk score) and patients in low risk group had longer survival time than high risk group (median OS 1074 vs. 372 days; P = 0.0033; Figure 2A).

We used the same β value obtained from the CGGA training set to calculate the risk score in two validation datasets. Patients with MGMT methylation were also divided into two groups, low risk group and high risk group, based on the median risk score. The CGGA (59 patients) and TCGA (55 patients) RNAseq datasets showed similar results (median OS 1074 vs. 372; P = 0.0001; Figure 2B; median OS 489 vs. 342; P = 0.0315; Figure 2A).

### Assessment of prognosis value of three-gene signature in related with chemotherapy

We evaluated the prognostic value of the signature for the GBM patients with MGMT promoter methylation. Furthermore, because MGMT promoter methylation status is highly associated with sensitivity to chemotherapy in GBM patients [6, 7], we assessed the predictive value of this signature for chemotherapy.

Twenty-one patients received recommended chemotherapy, while 17 patients didn't in the CGGA microarray dataset. Then we classified the 21 patients with three-gene signature into low and high risk group, and found a significant difference (P=0.0473) in OS as shown in Figure 2B. It also showed significant difference between low risk group with unmethylated group with chemotherapy (P=0.0197) (Figure 2B). Moreover, there is no significant difference between low and high risk group or unmethylated MGMT patients without chemotherapy (Figure 2B). We further validated the findings in the TCGA dataset (Figure 2C). The low risk group with chemotherapy treated showed the best outcomes, while the high risk group without chemotherapy treated showed the worst in combined analysis (Figure 2B, 2C). It turned out that the signature could specially recognize a group from the MGMT promoter methylation patients with chemotherapy treated, which showed similar outcomes to the patients with unmethylated MGMT promoter.

### Clinical and molecular features of low and high risk GBM patients with MGMT promoter methylation

**Figure 3:**
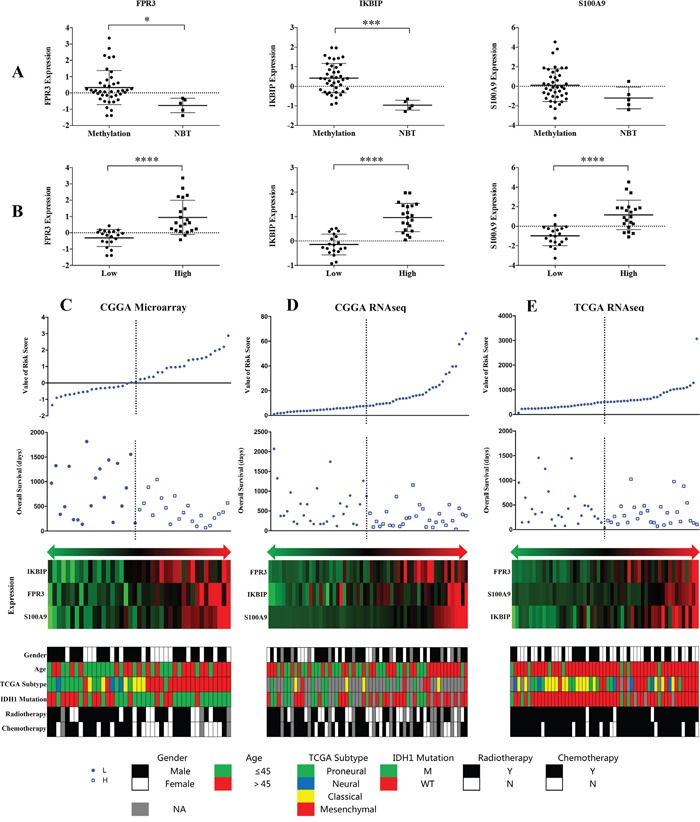
Distribution of risk score, OS, gene expression and clinical or molecular pathological features in CGGA microarray, RNA sequencing and TCGA RNA sequencing datasets

The expression levels of the three genes showed significant difference between patients with MGMT promoter methylation and non-cancerous brain tissue samples (Figure 3A). Moreover, this difference was also observed between low and high risk group (Figure 3B).

We assessed the independence of the three-gene signature in the CGGA microarray dataset. It showed that the signature was significant associated with the OS (P = 0.004) along with radiotherapy and chemotherapy in the univariate cox regression analysis. On multivariate analysis, it also showed the signature was an independent prognosis factor (P = 0.004) (Table 1). In TCGA and CGGA RNAseq dataset, the results indicated that the risk score was an independent prognostic factor (P = 0.007; P = 0.015) (Supplementary Table S2).

**Table 1:** Clinicopathologic factors associated with OS in the Cox regression analysis for patients from the CGGA microarray dataset

Variable	Univariate Cox		Multivariate Cox	
	p-value	HR	p-value	HR
Age	0.870	1.003		
Gender	0.740	0.883		
KPS	0.075	0.968		
IDH1	0.125	0.526		
Radiotherapy	0.001	0.242	0.007	0.264
Chemotherapy	0.002	0.292	0.217	0.565
Risk Score	0.004	1.643	0.004	2.195

Gender, male 1, female 2; IDH1 mutation status, mutated 1, wild-type 0; Radiotherapy, treated 1, untreated 0; Chemotherapy, treated 1, untreated 0.

We observed that GBM patients in the high risk group had shorter OS than low risk group (Figure 3C). The related clinical information such as gender, age, TCGA subtype, IDH1 mutation radiotherapy and chemotherapy were obtained from CGGA microarray database. Patients in high risk group tended to display older age (>45 years), classical and mesenchymal TCGA subtype and non-chemoradiotherapy. Moreover, we further validated in addition two datasets (Figure 3D, 3E).

### Functional annotation of the different prognosis

**Figure 4:**
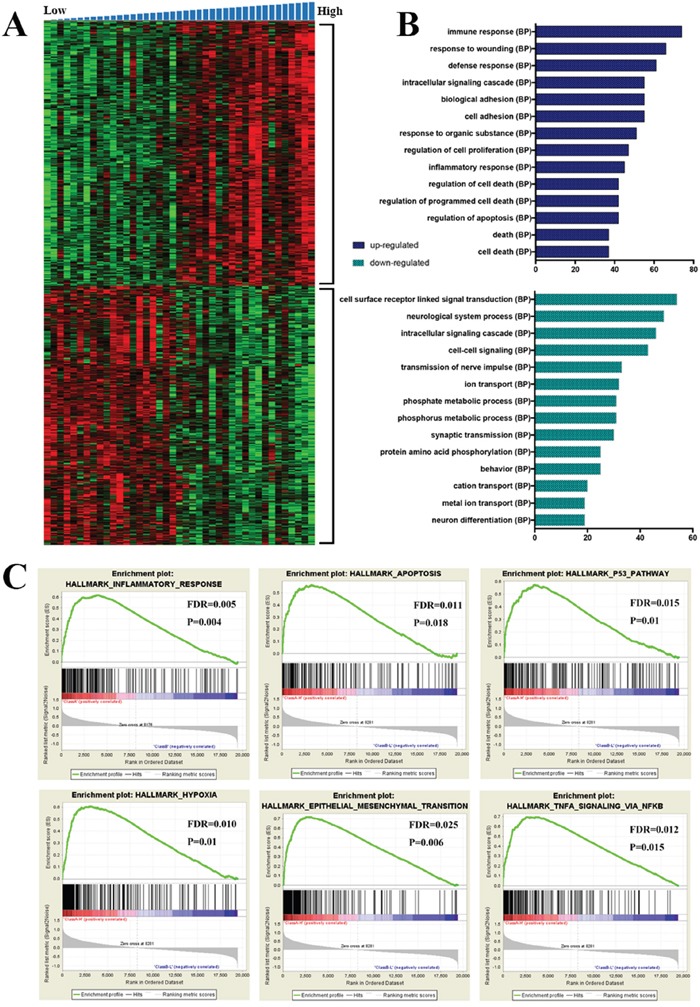
Functional annotation of each risk groups **A.** Hierarchical clustering analysis of mRNA expression profiles based on the top 1000 genes. **B.** Biological processes revealed the significant association of the genes with different expression in each group. Column length: gene counts. **C.** The top six enriched pathways in high risk group analyzed by gene set enrichment analysis.

We performed SAM (FDR < 0.05) between low and high risk group in the CGGA microarray dataset and we screened top 500 positively (599 probes) and 500 negatively (588 probes) correlated expression genes with the risk score, respectively. The expression patterns of genes were showed in Figure 4A using a hierarchical clustering analysis. Moreover, we performed the gene enrichment analysis using DAVID (The Database for Annotation, Visualization and Integrated Discovery) [8]. It showed that the biological processes, such as immune response, inflammatory response, regulation of cell death, regulation of apoptosis, biological adhesion and cell adhesion, et al (Figure 4B) were significantly enriched in the high risk group. In contrast, the biological processes, such as cell surface receptor linked signal transduction, neurological system process, intracellular signaling cascade and cell-cell signaling, et al (Figure 4B) were significantly enriched in low risk group. Gene enrichment analysis was further validated by Gene set enrichment analysis (GSEA) [9] and it revealed that the subgroup with high risk score had increased expression of inflammatory response, apoptosis, P53 pathway, hypoxia, epithelial mesenchymal transition (EMT) and TNFA signaling via NFKB (Figure 4C). Nuclear factor kB (NFKB) is a nuclear transcription factor that regulates expression of a large number of genes that are critical for the regulation of apoptosis, tumorigenesis, inflammation, and various autoimmune diseases [10–13].

Overall, the results of GSEA analysis were consistent with GO analysis' and functional annotation of the signature mainly enriched in immune response, apoptosis, cell adhesion pathways. It might partially explain the poor OS of patients with MGMT promoter methylation in high risk group.

## DISCUSSION

It is reported that the status of MGMT promoter methylation is closely associated with chemotherapy sensitivity [6, 7]. However, among the patients with methylation of MGMT, the overall survival is variance significantly. Therefore, we performed the analysis on the whole genome mRNA expression profiling to screen the determinant genes which can predict the overall survival of patients with equivalent MGMT methylated status. As far as we know, it is the first study for the type of patients.

The three genes have different expression between GBMs with non-cancerous tissues, and they all have prognosis values. The risk score based on the three genes divided patients into low and high risk groups and patients in low risk group had longer OS than high risk group. Further, low risk group also showed better OS than high risk groups and unmethylated group in patients with chemotherapy, but no significant difference between low and high or unmethylated MGMT patients without chemotherapy. The signature can specifically predict the prognosis of MGMT promoter methylation patients with chemotherapy treated. It also showed the signature was an independent prognostic factor along with age, gender, IDH1, radiotherapy and chemotherapy.

All of the 3 genes were remarkably associated with prognosis in GBM with MGMT methylation status. FPR3 is a member of the human formyl peptide receptors, which plays an important functional role in the regulation of immune responses and host defense mechanisms [14, 15]. FPR3 is also noted as FPRL2. It is reported that FPR3 can promote calcium mobilization and chemotaxis [16]. GO analysis showed that up-regulation genes were enriched in immune response and inflammatory response pathways. Moreover, it reported that immune response may play important role in GBM recently [17]. IKBIP (I kappa B kinase interacting protein) is a novel p53 target gene with proapoptotic function which is consistence with P53 pathway of GSEA results. It locates on chromosome 12 in close proximity to APAF1 (apoptotic protease-activating factor-1) and the two genes are transcribed in opposite directions [18]. Therefore, we considered it had similar functions to APAF1. APAF1 can encode a cytoplasmic protein that initiates apoptosis and it is a key regulator of mitochondrial apoptotic pathway. It is reported that decreased expression of APAF1 could be interpreted as an event contributing to melanoma chemo-resistance [19]. Moreover, APAF1 positively regulated the 5-FU-induced mitochondria-mediated apoptosis in colorectal cancer cells [20]. GO and GSEA analysis also confirmed that the three-gene signature may have a potential of regulation of apoptosis, cell death. S100A9 is a member of the S100 protein family. It has shown to regulate inflammatory processes [21] and be detected in various human cancers, such as breast cancer, prostate cancer and hepatocellular carcinoma [22–25]. It is reported that S100A9 promotes cell growth and invasion in hepatocellular carcinoma through stimulating MAPK signaling cascades [25]. GO analysis showed that related genes were enriched in biological adhesion and cell adhesion pathways, while EMT pathway in GSEA analysis. Moreover, S100A9 can induce inflammatory cytokines and is associated with overall survival in ER- PgR- breast cancers [22].

There are limitations in our manuscript, only 5 NBT samples were included into SAM analysis and only 41 patients (21 with chemotherapy; 17 non-chemotherapy) collected into our analysis which could cause bias of data analysis. The three-gene signature were selected based on the bioinformatics analysis and it is a totally observational study which may just provide clues for further study of GBM patients with MGMT promoter methylation. We will continue to collect samples and validated these findings experimentally in our future work. However, we further validated screened target genes in CGGA and TCGA RNAseq datasets.

In conclusion, our results showed that the three-gene signature has prognosis value for patients with MGMT promoter-methylated glioblastomas. Due to the poor prognosis in high risk group, clinicians should pay more attention to new treatment therapies. Further study need to validate these findings experimentally in future work.

## MATERIALS AND METHODS

### Patients and datasets

145 patients (106 microarray; 59 RNA sequencing) from the Chinese Glioma Genome Atlas (CGGA; http://www.cgga.org.cn/) were included in our analysis. All patients' clinical information was download from this website. The RNAseq data were normalized by log2 transformed before analysis. 119 patients from The Cancer Genome Atlas (TCGA; http://cancergenome.nih.gov/) were included as validation dataset.

### Signature development

We excluded patients without survival data or < 30 days due to they may die of other reasons. The risk score was developed based on a linear combination of the mRNA expression level (expr) weighted by the regression coefficient (β) derived from the univariate Cox regression analysis as previously reported [26–28]. The risk score for each patient was calculated as follows:

Risk score = β_gene1_ × expr_gene1_ + β_gene2_ × expr_gene2_+ ··· + β_genen_ × expr_genen_

The high risk score group presented shorter OS than low risk score group. The significance analysis of microarray (SAM) and Cox analysis was calculated using R software (version 3.2.3) with the samr and survival packages. Moreover, the univariate and multivariate cox regression analysis was performed by SPSS software (version 22; SPSS Inc., Chicago, IL, USA). The Kaplan-Meier analysis was used to evaluate the OS distributions by using GraphPad Prism 6 (GraphPad Software Inc., La Jolla, CA, USA). A two-sided P value of < 0.05 was regarded as statistically significant.

## SUPPLEMENTARY TABLES




