# Supplementary material for: A three-gene signature for prognosis in patients with MGMT promoter-methylated glioblastoma
Source: Oncotarget. 2016 Aug 31;7(43):69991–9. doi: 10.18632/oncotarget.11726 (PMC5342529; doi:10.18632/oncotarget.11726)
Supplement: Supplementary file 1 [file oncotarget-07-69991-s001.pdf]

A three-gene signature for prognosis in patients with MGMT promoter-methylated glioblastoma

SUPPLEMENTARY TABLES

Supplementary Table S1: Different expression genes between GBM with MGMT promoter methylation or unmethylation with non-cancerous brain tissue samples

See Supplementary File 1

Supplementary Table S2: Clinicopathologic factors associated with OS in the Cox regression analysis for patients from the CGGA RNAseq dataset and TCGA RNAseq dataset

CGGA RNAseq dataset

Variable	Univariate Cox		Multivariate Cox	
	p-value	HR	p-value	HR
Age	0.834	1.003		
Gender	0.410	0.728		
KPS	0.952	0.999		
IDH1	0.625	0.836		
Radiotherapy	0.001	0.250	0.003	0.288
Chemotherapy	0.023	0.411	0.036	0.432
Risk Score	0.013	1.022	0.015	1.026

Gender, male 1, female 2; IDH1 mutation status, mutated 1, wild-type 0; Radiotherapy, treated 1, untreated 0; Chemotherapy, treated 1, untreated 0.

TCGA RNAseq dataset

Variable	Univariate Cox		Multivariate Cox	
	p-value	HR	p-value	HR
Age	0.000	1.066	0.002	1.065
Gender	0.651	0.853		
IDH1	0.082	0.170		
Radiotherapy	0.021	0.232	0.694	0.732
Chemotherapy	0.036	0.418	0.701	0.825
Risk Score	0.005	1.001	0.007	1.001

Gender, male 1, female 2; IDH1 mutation status, mutated 1, wild-type 0; Radiotherapy, treated 1, untreated 0; Chemotherapy, treated 1, untreated 0.
